# Bibliography

**Published:** 1916-02

**Authors:** 


### BIBLIOGRAPHY

FOOD ANALYSIS. This is a work of about 500 pages, edited by Alpheus G. Woodman, S.B., of the Massachusetts Institute of Technology, and is published by the McGraw-Hill Book Co., of New York. It is a concise treatment of the typical methods used in food analysis. It is practically the course of instruction given by Professor Woodman to his classes. Naturally it is technical, but he takes great pains to interpret results and give the student data for discriminating judgment. It is a student's book rather than a practicing chemist's. The foods treated are fats and oils, carbohydrates, alcoholics, etc. Food colorings, chemical preservatives, milk, syrups, cocoa, spices, flavoring extracts are also carefully considered. While it may not be an important book for the average practitioner it will find a valued place in every scientific observer's library and laboratory.

